# Causal Relationships Between Pregnancy, Menstrual History, and Endometrial Cancer With Mediating Effects of Metabolism‐Related Traits

**DOI:** 10.1155/humu/3401957

**Published:** 2025-12-18

**Authors:** Meifang Zhou, Suiping Dai, Tingting Zhu, Shihao Hong

**Affiliations:** ^1^ Assisted Reproduction Unit, Department of Obstetrics and Gynecology, Sir Run Run Shaw Hospital, School of Medicine, Zhejiang University, Hangzhou, China, zju.edu.cn; ^2^ Zhejiang Provincial Clinical Research Center for Reproductive Health and Disease, Hangzhou, 310016, China; ^3^ Department of Obstetrics and Gynecology, Quanzhou Hospital of Traditional Chinese Medicine, Quanzhou, China

**Keywords:** endometrial cancer, mediator, Mendelian randomization, periods, pregnancy

## Abstract

**Background:**

Periods and pregnancy may affect the development of endometrial cancer by affecting the secretion of sex hormones, but the causal relationship is not clear, and its mediating factors need to be explored.

**Methods:**

In this study, multivariable Mendelian randomization was used to analyze summary statistics of genome‐wide association studies of European ancestry, to evaluate the effect of 10 period‐ or pregnancy‐related factors on endometrial cancer. In addition, we performed the heterogeneity test and pleiotropy test to analyze the sensitivity. Because of the effect of sex hormones on body metabolism and the relationship between metabolism‐related traits and cancer, we explored the mediating effect of metabolism‐related traits by two‐step Mendelian randomization.

**Results:**

This study showed that age at menarche (*p* = 1.21e − 05; OR = 0.6852; 95% CI: 0.5784–0.8116), age at menopause (*p* = 0.00098; OR = 1.242; 95% CI: 1.0919–1.4127), and sex hormone–binding globulin (SHBG) levels (*p* = 7.4e − 07; OR = 0.5914; 95% CI: 0.4804–0.7281) have an independent causal relationship with the incidence of endometrial cancer. Moreover, several obesity‐related traits play a mediating role in the causal relationship between age at menarche and endometrial cancer. The mediators and their mediating effects are BMI (55.54%), obesity (30.37%), waist circumference preference (27.67%), body fat percentage (17.61%), and waist‐to‐hip ratio (14.82%). These results are robust to sensitivity analysis.

**Conclusion:**

This study demonstrated the independent effect of pregnancy‐ and period‐related factors on endometrial cancer and suggested that avoiding obesity may be an effective method to prevent endometrial cancer for patients with premature menarche.

## 1. Introduction

Endometrial cancer represents the most frequently diagnosed gynecological cancer in high‐income countries [1]. As of 2020, the worldwide burden of this disease has amounted to 417,367 newly identified cases and 97,370 deaths attributable to endometrial cancer [2]. Despite the decreasing incidence of various cancer types over the last 20 years, endometrial cancer incidence rates continue to rise worldwide [3–5]. However, the pathogenesis of endometrial carcinoma is still unclear. Pregnancy involves prolonged exposure to high levels of progesterone, which may have a protective effect against endometrial hyperplasia and cancer by counteracting estrogen‐induced proliferation. Factors like the number of full‐term pregnancies and age at last birth are thus thought to be inversely associated with endometrial cancer risk [6, 7], but the causal effect and mediators still need to be explored.

Mendelian randomization (MR) is a powerful analytical approach that leverages germline genetic variants as instrumental variables (IVs) for risk factors to assess the causal effects of these factors on disease outcomes in observational settings [8]. Given the random assortment of germline genetic variants during meiosis, MR analyses may be less susceptible to confounding by lifestyle and environmental factors compared to conventional observational studies. Moreover, as germline genetic variants are established at conception and do not change over time, MR analyses are less prone to reverse causation bias [9]. In addition, two‐step MR analysis can identify the mediators in a causal relationship between exposures and outcomes [10].

In this study, two‐sample multivariable MR (MVMR) was used to investigate the independent causal associations of pregnancy‐ and period‐related factors with endometrial cancer. Besides, we further performed a two‐step MR to explore the mediator effect of metabolism‐related traits in the causal effect between the period‐ and pregnancy‐related exposures and endometrial cancer. The specific pregnancy‐related factors chosen as exposures (e.g., gestational diabetes and gestational hypertension) were selected based on their established associations in prior epidemiological studies and availability of large, well‐powered GWAS summary statistics for MR analysis. We discovered that there is an independent causal relationship between age at menopause, age at menarche, and sex hormone–binding globulin (SHBG) levels and endometrial cancer. Moreover, the obesity‐related traits play an important role in the causal relationship between age at menarche and endometrial cancer. These results are of great significance for the prevention and treatment of endometrial cancer.

## 2. Methods

### 2.1. Study Design

This study was divided into two stages (Figure 1a). In the first stage, we utilized the univariable MR (UVMR) and MVMR methods to evaluate the causal relationship between pregnancy‐ and period‐related factors with endometrial cancer. Single‐nucleotide polymorphisms (SNPs) were used as IVs to represent the exposures, and the causal relationship between the exposures and the outcome was further investigated. The UVMR results showed that three period‐related factors, including age at menopause, age at menarche, and SHBG levels, were causally related to endometrial cancer, while no causal relationship was found between the pregnancy‐related factors included in this study and endometrial cancer. Therefore, we further analyzed the causal relationship between the three exposures (age at menopause, age at menopause menarche, and SHBG levels) and endometrial cancer using the MVMR method, and the results showed that there was still an independent causal relationship between the three variables and endometrial cancer after adjusting for each other.

Figure 1Overview of the study design. (a) Study design. This study consisted of two stages. In Stage 1, we used two‐sample multivariable Mendelian randomization (MVMR) to analyze the causal relationship between pregnancy‐ and period‐related factors and endometrial cancer. In Stage 2, we used two‐step MR to explore the mediating effects of metabolic factors. (b) Mediator selection process in Stage 2. During the mediator selection process in Stage 2, we first used univariable Mendelian randomization (UVMR) to identify mediators that were causally related to the exposure. Then, we used MVMR to identify mediators that were also causally related to endometrial cancer after adjusting for the corresponding exposures.

**Figure:**
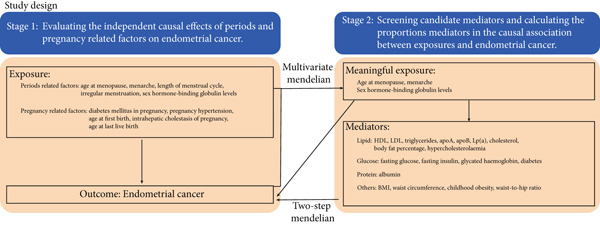
(a)

**Figure:**
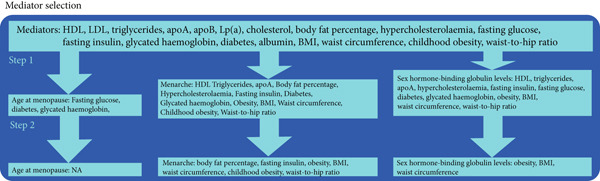
(b)

In the second stage, we used the two‐step MR to explore the intermediate variables that mediate the effect of age at menopause, age at menarche, and SHBG levels on endometrial cancer. Based on previous studies, we analyzed the mediating effects of 19 variables related to metabolism. The results of the two‐step MR showed that body fat percentage, obesity, BMI, waist circumference, and waist‐to‐hip ratio played a mediating role in the effect of age at menarche on endometrial cancer. This study is reported according to the Strengthening the Reporting of Observational Studies in Epidemiology Using Mendelian Randomization (STROBE‐MR) guideline [11].

### 2.2. Data Sources

In this study, all data were summary‐level data from genome‐wide association studies (GWASs) conducted in people of European ancestry (Table 1). Specifically, the data included in this study were as follows.

**Table 1:** Characteristics of contributing studies.

**Phenotype**	**Population**	**Participants**	**Data source**	**Year of publication**	**PMID**
Exposure					
Diabetes mellitus in pregnancy	European	6033 cases and 110,330 controls	FinnGen	2021	NA
Pregnancy hypertension	European	7686 cases and 115,893 controls	FinnGen	2021	NA
Age at first birth	European	542,901	Meta	2021	34211149
Age at menopause	European	143,819	UK Biobank	2018	NA
Age when periods started (menarche)	European	243,944	UK Biobank	2018	NA
Age at last live birth	European	170,248	UK Biobank	2018	NA
Intrahepatic cholestasis of pregnancy (ICP)	European	940 cases and 122,639 controls	FinnGen	2021	NA
Length of menstrual cycle	European	43,125	UK Biobank	2018	NA
Excessive, frequent, and irregular menstruation	European	17,873 cases and 68,969 controls	FinnGen	2021	NA
Sex hormone–binding globulin levels	European	370,125	UK Biobank	2020	32042192
Outcome					
Endometrial cancer	European	8758 cases and 46,126 controls	Meta	2018	30093612
Mediators					
HDL cholesterol	European	403,943	UK Biobank	2020	32203549
LDL cholesterol	European	440,546	UK Biobank	2020	32203549
Triglycerides	European	441,016	UK Biobank	2020	32203549
Apolipoprotein A	European	393,193	UK Biobank	2020	32203549
Apolipoprotein B	European	439,214	UK Biobank	2020	32203549
Lipoprotein A	European	377,572	UK Biobank	2018	NA
Serum total cholesterol	European	21,491	Meta	2016	27005778
Body fat percentage	European	454,633	UK Biobank	2018	NA
Hypercholesterolemia	European	22,622 cases and 440,388 controls	UK Biobank	2018	NA
Fasting insulin	European	151,013	Meta	2021	34059833
Fasting glucose	European	200,622	Meta	2021	34059833
Diabetes	European	18,228 cases and 444,705 controls	UK Biobank	2018	NA
Glycated hemoglobin	European	467,800	UK Biobank	2018	NA
Albumin	European	432,239	UK Biobank	2018	NA
Obesity	European	4688 cases and 458,322 controls	UK Biobank	2018	NA
Body mass index (BMI)	European	461,460	UK Biobank	2018	NA
Waist circumference	European	462,166	UK Biobank	2018	NA
Childhood obesity	European	5530 cases and 8318 controls	Meta	2012	22484627
Waist‐to‐hip ratio	European	212,244	Meta	2015	25673412

### 2.3. Exposures

This study included 10 exposure factors, all of which were related to pregnancy or periods [12–14]. These 10 GWAS datasets were primarily derived from either the UK Biobank (UKB) or the FinnGen study [15]. The UKB is a prospective cohort of over 500,000 individuals aged 40–69 recruited from the general population in the United Kingdom, which includes over 200,000 women [16]. The UKB incorporates data from 143,819 women on age at menopause, 243,944 women on age at menarche, 170,248 women on age at last live birth, 43,125 women on menstrual cycle length, and 370,125 women on SHBG levels. The FinnGen study is a nationwide GWAS study from Finland that is linked to longitudinal phenotype and national health registry‐generated digital health record data and has little overlap with exposure or mediator GWASs [15]. The FinnGen study included 6033 cases of gestational diabetes, 7686 cases of gestational hypertension, 940 cases of intrahepatic cholestasis of pregnancy (ICP), and 17,873 cases of excessive, frequent, and irregular menstruation.

### 2.4. Mediators

Based on previous research, we explored the mediating effects of metabolic factors on the relationship between periods and endometrial cancer. The mediators included (1) lipid‐related traits (HDL, LDL, triglycerides, apoA, apoB, Lp[a], cholesterol, body fat percentage, and hypercholesterolemia), (2) glucose‐related traits (fasting glucose, fasting insulin, glycated hemoglobin, and diabetes), (3) protein‐related traits (albumin), and (4) adiposity traits (BMI, waist circumference preference, childhood obesity, waist‐to‐hip ratio, and obesity). The GWAS data for these mediators mainly came from the UKB or other large meta‐analyses [17–20]. Subsequently, we screened these mediators (Figure 1b). First, we used UVMR to select mediators that were causally related to age at menopause, menarche, and SHBG levels, and for which exposure had an effect on mediators. Next, we used MVMR to identify mediators that still had a causal effect on endometrial cancer even after removing the effect of exposures, and we further calculated their mediation effects. Sample overlap between the exposure, mediator, and outcome GWAS was minimal, as they were predominantly sourced from independent consortia. Any potential overlap would bias estimates toward the null under the weak instrument bias, but our high *F*‐statistics (> 10) mitigate this concern. We acknowledge that the winner′s curse in the discovery of GWAS may inflate instrument strength, though the use of strict significance thresholds helps to minimize this issue.

### 2.5. Outcome

In this study, we employed the outcome dataset from a meta‐GWAS for endometrial cancer, which was published by O′Mara et al. [21]. This dataset included a total of 8758 endometrial cancer cases and 46,126 controls. This dataset was derived from 13 endometrial cancer studies. All participants were of European ancestry.

### 2.6. Statistical Analysis

#### 2.6.1. UVMR and MVMR Analyses

In this study, we used UVMR to assess the effects of 10 exposures related to pregnancy or periods on endometrial cancer. We then performed MVMR to evaluate the independent effects of these exposures on endometrial cancer. All MR analyses met three key assumptions: (1) In UVMR analysis, genetic variation is closely associated with exposure; in MVMR analysis, genetic variation is closely associated with at least one of the multiple exposures; (2) genetic variation is not associated with confounding factors that affect the relationship between exposure and endometrial cancer; and (3) the instruments used affect endometrial cancer through the exposure and not solely through other factors [22]. For the two‐step mediation analysis, additional assumptions include (1) no confounding of the mediator–outcome relationship by the exposure, (2) no exposure‐induced mediator–outcome confounding, and (3) the absence of mediator–outcome confounders affected by the exposure. We note that the proportion mediated is an approximation derived from odds ratios (ORs), which are noncollapsible, and that estimates can be biased by differences in instrument strength between the two steps of the analysis.

To meet these assumptions, we selected SNPs closely related to exposure (*p* < 5 × 10^−8^), used linkage disequilibrium analysis (*r* ^2^ < 0.001; distance threshold, 10,000 kb) to screen SNPs, and finally selected SNPs with an *F*‐value > 10 as IVs. We used inverse variance weighted (IVW) as the main UVMR and MVMR method, which combines the Wald ratio estimates of each SNP into a single causal estimate for the corresponding exposure using a random effects meta‐analysis. The same covariate set was used for adjustment in the two samples.

#### 2.6.2. Mediation MR Analyses

We conducted a two‐step MR to assess whether 19 metabolic factors act as intermediate factors in the relationship between these exposures and endometrial cancer [23]. In Stage 1, we used UVMR to evaluate the causal effects (*β*1) of these three exposures on mediators. In Stage 2, we assessed the causal effects of mediators (*β*2) on endometrial cancer and adjusted for the corresponding exposure using MVMR.

In Stage 1, we obtained the causal effect of exposure on the outcome (*β*0). If *β*0, *β*1, and *β*2 are all significant (*p* < 0.05), we consider that the exposure is causally related to endometrial cancer and a part of this association is mediated by the intermediate variables. For exposures and mediators with significant *β*0, *β*1, and *β*2, we calculated the proportion of the exposure effect on endometrial cancer mediated by mediators. We multiplied the results of the two steps in the two‐step MR to obtain the mediated effect of the mediator (*β*1∗*β*2) and calculated *β*1∗*β*2/*β*0 as the proportion of the exposure effect on endometrial cancer mediated by mediators [24]. We used the delta method to derive the standard error (SE) using effect estimates obtained from the two‐sample MR analysis [25].

#### 2.6.3. Sensitivity Analyses

We employed weighted median, MR Egger, and MR pleiotropy residual sum and outlier methods (MRS) to assess the robustness of IVW results in UVMR analysis [26] and MVMR Egger to validate IVW results in MVMR analysis. We used MR Egger′s intercept to test for horizontal pleiotropy, and a large difference from zero (*p* < 0.05) indicated its presence [27, 28]. We also used Cochran′s *Q* statistic for MR Egger and IVW to evaluate heterogeneity between instruments, and a *p* value less than 0.05 indicated the possibility of heterogeneity [29]. For exposures with heterogeneity, we used the random effects model to estimate MR effects [30], and a *p* value less than 0.05 indicated a causal relationship. Additionally, we conducted “leave‐one‐out” sensitivity analysis to demonstrate that the causal effects of age at menopause, age at menarche, and SHBG levels on endometrial cancer were not affected by single SNPs [31]. We used conditional *F*‐statistics to test instrument validity, with *F* < 10 indicating low instrument validity [32]. We used publicly available GWAS summary statistics with high imputation quality and low missing rates; thus, no additional missing data processing was required.

Effect sizes in this study were represented by OR or *β* coefficient, and the corresponding 95% CI were displayed. All analyses in this study were performed using the “TwoSampleMR,” “MRPRESSO” [33], “MendelianRandomization” [34], “MVMR,” “forestplot,” and “ggplot2” [35] packages in R software (Version 4.2.0).

## 3. Results

### 3.1. Univariate and Multivariate MR Estimates of the Causal Associations of Pregnancy‐ and Period‐Related Factors With Endometrial Cancer

In this study, we conducted UVMR analysis to investigate the causal relationship between 10 exposures related to pregnancy and periods and endometrial cancer. As shown in Figure 2a, we found that age at menopause, age at menarche, and SHBG levels were causally associated with endometrial cancer (*p* < 0.05), with respective SNPs of 107, 194, and 378 and *F*‐values of 10.88, 19.15, and 24.5. Effect estimates for age at menarche and age at menopause are expressed per 1‐year increase and, for SHBG, per 1 standard deviation (SD) increase (approximately 30.3 nmol/L). For instance, a 1‐year increase in genetically predicted age at menarche was associated with an approximately 32% lower risk of endometrial cancer (OR = 0.68). However, no causal relationship was found between the five exposures related to pregnancy and endometrial cancer. For period‐related factors, age at menopause was positively associated with endometrial cancer incidence (*p* = 0.00098; OR = 1.242; 95% CI: 1.0919–1.4127), while age at menarche and SHBG levels were negatively associated with endometrial cancer incidence (*p* = 1.21e − 05; OR = 0.6852; 95% CI: 0.5784–0.8116 and *p* = 7.4e − 07; OR = 0.5914; 95% CI: 0.4804–0.7281, respectively). To test the independent causal relationship between the three variables and endometrial cancer, multivariate Mendelian random analysis was performed. And we discovered that age at menopause, age at menarche, and SHBG levels were causally associated with endometrial cancer even after removing the effects of the other two (Figure 2b). Our genetic instruments for age at menarche and menopause proxy lifelong predispositions and may not capture nonlinear effects or critical windows of risk. Future research applying nonlinear MR methods or conducting age‐stratified analyses could help identify the most pertinent periods for intervention.

Figure 2The results of UVMR and MVMR. (a) The causal association of period‐ and pregnancy‐related factors with endometrial cancer in an univariable inverse variance weighted model. (b) The causal association of age at menarche, age at menopause, and SHBG with endometrial cancer in a multivariable inverse variance weighted model. UVMR, univariable Mendelian randomization; MVMR, multivariable Mendelian randomization; CI, confidence interval; OR, odds ratio; SHBG, sex hormone–binding globulin; SE, standard error.

**Figure:**
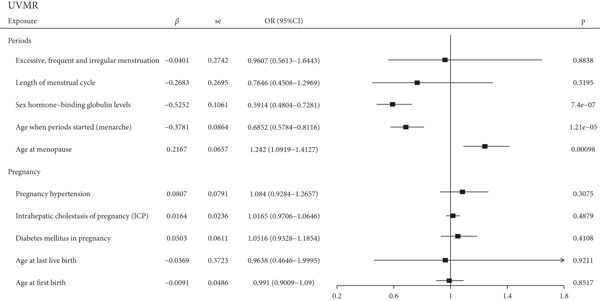
(a)

**Figure:**
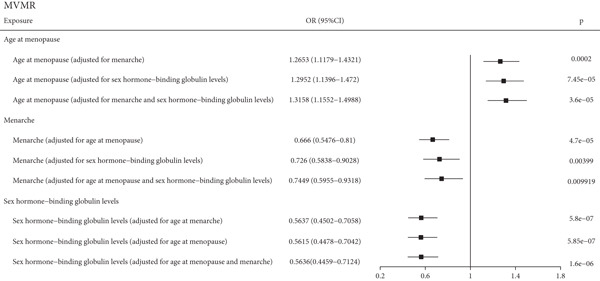
(b)

To validate the results, we used MR Egger, weighted median, simple mode, and weighted mode (Supporting Information 1: Table S1). Sensitivity analysis, including heterogeneity test, pleiotropy test, and leave‐one‐out sensitivity test, was performed on the UVMR results (Supporting Information 2: Table S2). We assessed the heterogeneity between instruments using the Cochran′s *Q* statistic of MR Egger and IVW, which showed heterogeneity for age at menopause, age at menarche, and SHBG levels (*p* < 0.05, Figures 3a, 3b, and 3c). Therefore, we used a random effects model to estimate the MR effect sizes, which confirmed the causal relationship between these exposures and endometrial cancer (*p* < 0.05). We also used MR Egger′s intercept to test for horizontal pleiotropy and found no significant difference from zero (*p* > 0.05, Figures 3d, 3e, and 3f), indicating the absence of horizontal pleiotropy.

Figure 3The results of the sensitivity analysis. (a–c) The scatter diagrams show the results of inverse variance weighted, MR Egger, simple mode, weighted median, and weighted mode analyses. Each point on the scatter diagrams represents an instrumental variable (IV), and the line passing through each point reflects the 95% confidence interval (CI). The horizontal axis represents the effect of SNPs on three exposures, while the vertical axis represents their effect on endometrial cancer. The colored lines represent the MR fitting results. (d–f) These three funnel plots display the heterogeneity of SNPs, and overall, the three plots are symmetric.

**Figure:**
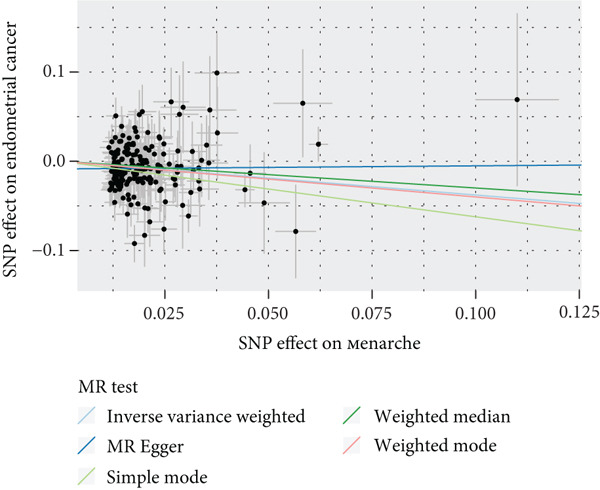
(a)

**Figure:**
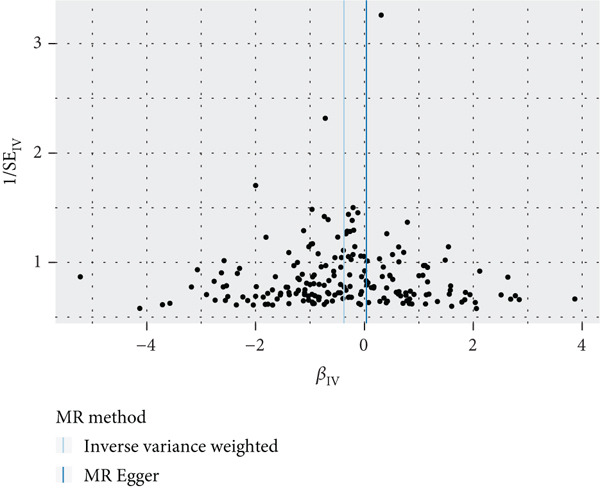
(b)

**Figure:**
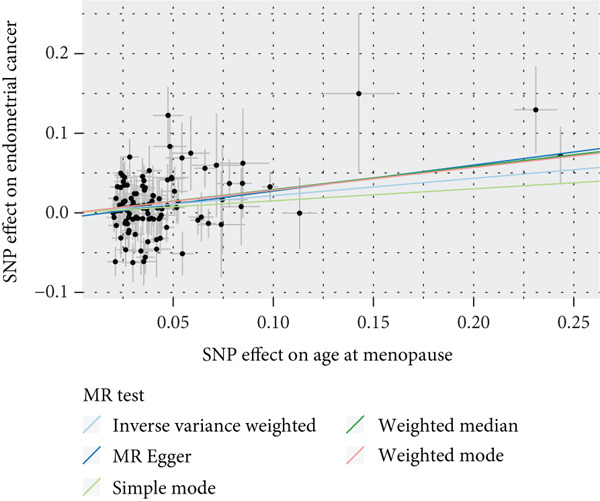
(c)

**Figure:**
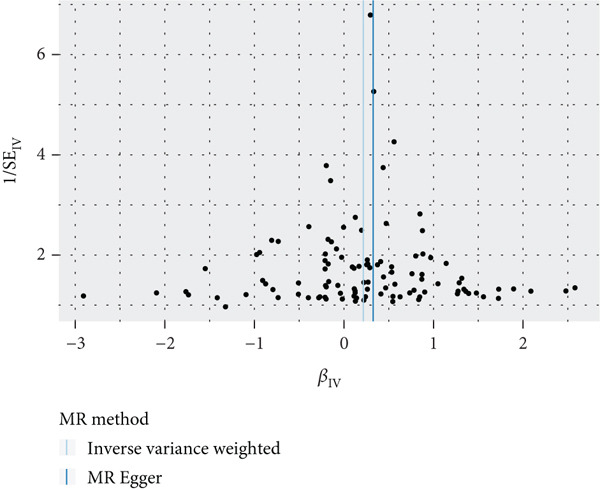
(d)

**Figure:**
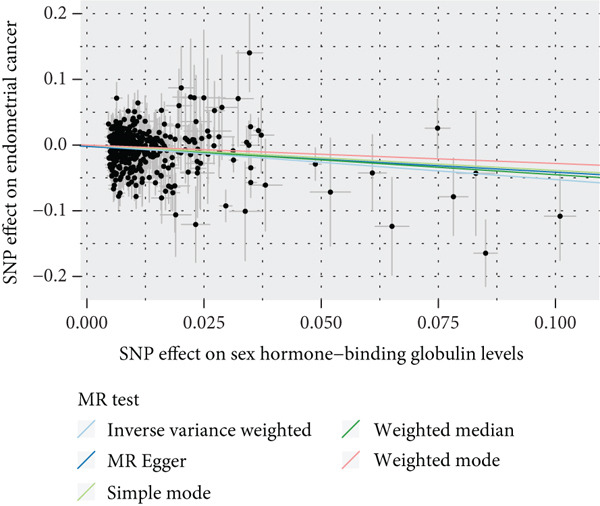
(e)

**Figure:**
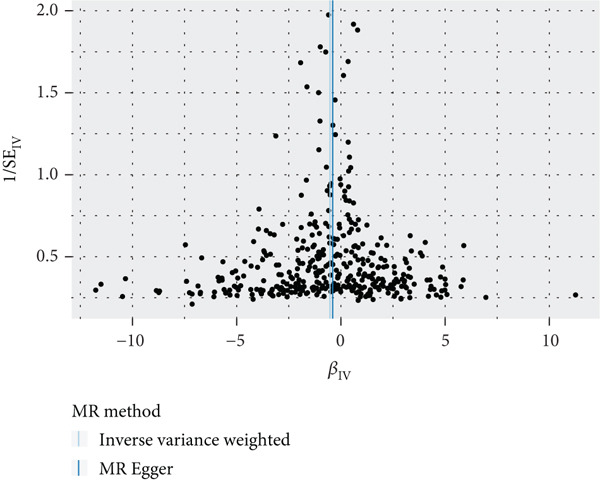
(f)

### 3.2. Effect of Exposures on Mediators

In this study, we screened 19 candidate mediators using two‐step MR. In the Step 1 UVMR analysis (Table 2), we found negative correlations between fasting glucose, diabetes, and glycated hemoglobin with age at menopause. We also found positive correlations between HDL, apoA, and fasting insulin with age at menarche and negative correlations between triglycerides, body fat percentage, hypercholesterolemia, diabetes, glycated hemoglobin, obesity, BMI, waist circumference, childhood obesity, and waist‐to‐hip ratio with age at menarche. Additionally, we found that SHBG levels were positively correlated with HDL and apoA and negatively correlated with triglycerides, hypercholesterolemia, fasting insulin, fasting glucose, diabetes, glycated hemoglobin, obesity, BMI, waist circumference, and waist‐to‐hip ratio. We also performed sensitivity analyses using the heterogeneity test, random effects model, pleiotropy test, and leave‐one‐out sensitivity test (Supporting Information 2: Table S2). Despite strong heterogeneity, the causal relationships between these mediators and exposures are confirmed (random effects model: *p* < 0.05). In the horizontal pleiotropy analysis, we found that the effects of SHBG levels on obesity, BMI, and waist circumference, and the effects of age at menarche on childhood obesity, had horizontal pleiotropy (*p* < 0.05).

**Table 2:** UVMR assessing the causal association between exposures and mediators.

**Exposures**	**Mediators**	**nSNP**	**β** **(95% CI)**	**OR (95% CI)**	**p** **value**
Age at menopause	Fasting glucose	107	−0.0154 (−0.0227 to −0.0081)	0.9847 (0.7969 to 0.9919)	0.0454
	Diabetes	107	−0.0038 (−0.0305 to −0.0003)	0.9962 (0.9700 to 0.9997)	0.0285
	Glycated hemoglobin	107	−0.0673 (−0.0835 to −0.0516)	0.9349 (0.9199 to 0.9497)	7.77e − 05

Menarche	HDL	182	0.0850 (0.0651 to 0.1049)	1.0887 (1.0673 to 1.1106)	4.69e − 05
	Triglycerides	182	−0.0817 (−0.1025 to −0.0609)	0.9216 (0.9026 to 0.9409)	0.0002
	ApoA	182	0.0719 (0.0537 to 0.0901)	1.0746 (1.0552 to 1.0943)	0.0002
	Body fat percentage	196	−0.1178 (−0.1442 to −0.0914)	0.8889 (0.8657 to 0.9127)	2.21e − 05
	Hypercholesterolemia	195	−0.0077 (−0.01 to −0.0054)	0.9923 (0.9900 to 0.9976)	0.0014
	Fasting insulin	191	0.0308 (0.0192 to 0.0424)	1.0313 (1.0194 to 1.0433)	0.0113
	Diabetes	195	−0.0164 (−0.0192 to −0.0136)	0.9838 (0.9818 to 0.9865)	2.51e − 08
	Glycated hemoglobin	196	−0.0856 (−0.1054 to −0.0658)	0.9179 (0.9000 to 0.9363)	3.96e − 05
	Obesity	180	−0.0064 (−0.0076 to −0.0052)	0.9936 (0.9924 to 0.9948)	8.56e − 07
	BMI	196	−0.3473 (−0.3865 to −0.3081)	0.7066 (0.6794 to 0.7348)	3.89e − 17
	Waist circumference	196	−0.1746 (−0.2079 to −0.1414)	0.8398 (0.8123 to 0.8681)	6.14e − 07
	Childhood obesity	92	−0.9590 (−1.2051 to −0.713)	0.3833 (0.2997 to 0.4902)	0.0002
	Waist‐to‐hip ratio	99	−0.1009 (−0.1429 to −0.0589)	0.904 (0.8668 to 0.9428)	0.0224

Sex hormone–binding globulin levels	HDL	342	0.4902 (0.4429 to 0.5375)	1.6326 (1.5572 to 1.7117)	7.25e − 23
	Triglycerides	342	−0.576 (−0.6355 to −0.5165)	0.5621 (0.3648 to 0.4838)	3.64e − 20
	ApoA	342	0.3736 (0.3271 to 0.4201)	1.4529 (1.3571 to 1.5487)	2.11e − 14
	Hypercholesterolemia	322	−0.024 (−0.0278 to −0.0202)	0.9763 (0.9726 to 0.9800)	2.25e − 09
	Fasting insulin	354	−0.0849 (−0.1087 to −0.0612)	0.9186 (0.8970 to 0.9406)	0.0007
	Fasting glucose	357	−0.0594 (−0.0834 to −0.0354)	0.9424 (0.9200 to 0.9681)	0.019
	Diabetes	320	−0.0314 (−0.036 to −0.0268)	0.9691 (0.9646 to 0.9736)	6.9e − 11
	Glycated hemoglobin	379	−0.2758 (−0.3161 to −0.2355)	0.7590 (0.7290 to 0.7928)	7.51e − 11
	Obesity	273	−0.0048 (−0.0063 to −0.0033)	0.9952 (0.9937 to 0.9967)	0.0028
	BMI	326	−0.1687 (−0.2089 to −0.1285)	0.8448 (0.8115 to 0.8794)	0.0001
	Waist circumference	326	−0.1443 (−0.1771 to −0.1115)	0.8657 (0.8377 to 0.8945)	2.83e − 05
	Waist‐to‐hip ratio	115	−0.2342 (−0.3035 to −0.1649)	0.7912 (0.7382 to 0.8480)	0.0013

### 3.3. Effect of Mediators on Endometrial Cancer With Adjustment for Exposures

**Figure 4:**
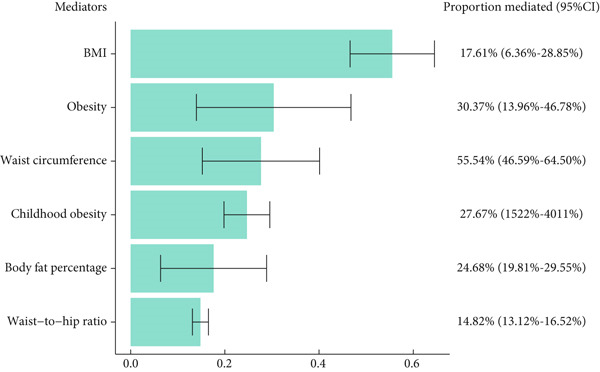
Mendelian randomization (MR) estimates of proportions mediated by mediators in the causal association between age at menarche and endometrial cancer.

In the second step of the two‐step MR, we examined the causal effects between candidate mediators and endometrial cancer, adjusted for exposures (Table 3). Using MVMR, we found that all three mediators related to age at menopause were not causally related to endometrial cancer. Among the mediators related to age at menarche, body fat percentage, obesity, BMI, waist circumference, childhood obesity, and waist‐to‐hip ratio were independently causally related to endometrial cancer. Among the mediators related to SHBG levels, obesity, BMI, and waist circumference were independently causally related to endometrial cancer. Based on the results of horizontal pleiotropy in the first step, we identified that body fat percentage, obesity, waist‐to‐hip ratio, BMI, and waist circumference—the five obesity‐related traits—mediate the causal relationship between age at menarche and endometrial cancer, with the proportion of the total causal effect mediated by each factor and the corresponding 95% confidence interval being 17.61% (6.36%–28.85%), 30.37% (13.96%–46.78%), 55.54% (46.59%–64.50%), 27.67% (15.22%–40.11%), and 14.82% (13.12%–16.52%) (Figure 4). Candidate mediators for SHBG levels and age at menopause were both excluded after two‐step MR analysis.

**Table 3:** MVMR assessing the causal association between mediators and endometrial cancer with adjustment for exposures.

**Exposures**	**Mediators**	**nSNP**	**β** **(95% CI)**	**OR (95% CI)**	**p** **value**
Age at menopause	Fasting glucose	49	−0.0318 (−0.3433 to 0.2797)	0.9687 (0.7094 to 1.3227)	0.8414
	Diabetes	48	0.5122 (−1.4178 to 2.4422)	1.669 (0.2422 to 11.4983)	0.6029
	Glycated hemoglobin	227	−0.0077 (−0.1043 to 0.0889)	0.9923 (0.901 to 1.093)	0.876

Menarche	HDL	260	0.0158 (−0.0993 to 0.1309)	1.0159 (0.9055 to 1.1399)	0.7876
	Triglycerides	222	0.0012 (−0.1071 to 0.1095)	1.0012 (0.8984 to 1.1157)	0.9828
	ApoA	213	0.0405 (−0.0742 to 0.1552)	1.0413 (0.9285 to 1.1679)	0.489
	Body fat percentage	282	0.5651 (0.3664 to 0.7639)	1.7596 (1.4425 to 2.1466)	2.49e − 08
	Hypercholesterolemia	14	−0.7446 (−2.9422 to 1.453)	0.4749 (0.0527 to 4.2759)	0.5066
	Fasting insulin	22	0.4253 (−0.0879 to 0.9384)	1.53 (0.9159 to 2.5559)	0.1043
	Diabetes	45	−0.0314 (−1.8098 to 1.747)	0.9691 (0.1637 to 5.7374)	0.9724
	Glycated hemoglobin	221	0.0144 (−0.0778 to 0.1065)	1.0145 (0.9251 to 1.1124)	0.7599
	Obesity	2	1.7935 (0.8786 to 2.7084)	6.0105 (2.4075 to 15.0052)	0.0001
	BMI	335	0.6047 (0.464 to 0.7453)	1.8307 (1.5904 to 2.1071)	3.61e − 17
	Waist circumference	257	0.7234 (0.5522 to 0.8946)	2.0614 (1.7371 to 2.4464)	1.22e − 16
	Childhood obesity	4	0.0973 (0.0241 to 0.1705)	1.1022 (1.0244 to 1.1859)	0.0092
	Waist‐to‐hip ratio	17	0.5564 (0.251 to 0.8617)	1.7444 (1.2853 to 2.3672)	0.0004

Sex hormone–binding globulin levels	HDL	237	0.0214 (−0.0955 to 0.1382)	1.0216 (0.9089 to 1.1482)	0.72
	Triglycerides	193	−0.0749 (−0.1908 to 0.041)	0.9278 (0.8263 to 1.0419)	0.2055
	ApoA	192	0.0349 (−0.0808 to 0.1507)	1.0355 (0.9224 to 1.1626)	0.554
	Hypercholesterolemia	8	−1.5038 (−4.0862 to 1.0786)	0.2223 (0.0168 to 2.9406)	0.2537
	Fasting insulin	16	0.3833 (−0.0686 to 0.8351)	1.4671 (0.9337 to 2.305)	0.0964
	Fasting glucose	29	0.0015 (−0.2839 to 0.2869)	1.0015 (0.7528 to 1.3323)	0.9919
	Diabetes	37	0.8479 (−0.8773 to 2.5731)	2.3347 (0.4159 to 13.1064)	0.3354
	Glycated hemoglobin	186	−0.0194 (−0.1151 to 0.0763)	0.9808 (0.8913 to 1.0793)	0.6909
	Obesity	1	1.5369 (0.6469 to 2.4268)	4.6502 (1.9096 to 11.3226)	0.0203
	BMI	259	0.5105 (0.3625 to 0.6584)	1.6661 (1.4369 to 1.9317)	1.35e − 11
	Waist circumference	192	0.6081 (0.4157 to 0.8005)	1.8369 (1.5154 to 2.2267)	5.86e − 10
	Waist‐to‐hip ratio	10	0.2887 (−0.063 to 0.6404)	1.3347 (0.9389 to 1.8972)	0.1076

## 4. Discussion

Previous studies have shown that periods and pregnancy factors are closely related to endometrial cancer [36–38], possibly due to the changes in estrogen and progesterone levels during menstrual or pregnancy cycles [39], but their causal relationships are not yet clear. This study provides new evidence for the effects of menstrual and pregnancy factors on endometrial cancer using MR. Our findings showed that for every 1‐year increase in age at menarche, the risk of endometrial cancer decreases by approximately 32%; for every 1 SD (about 30.3 nmol/L) increase in SHBG levels, the risk of endometrial cancer decreases by approximately 41%; and for every 1‐year increase in age at menopause, the risk of endometrial cancer decreases by approximately 24%. We further investigated the potential mediators between these three exposures and endometrial cancer and identified five mediators from 19 metabolic factors related to age at menarche and endometrial cancer. These mediators were ranked by their mediation proportion, including BMI (55.54%), obesity (30.37%), waist circumference (27.67%), body fat percentage (17.61%), and waist‐to‐hip ratio (14.82%). Our results demonstrated the independent effects of age at menarche, age at menopause, and SHBG levels on endometrial cancer and also highlighted the important mediating role of several common metabolic factors, mainly obesity‐related factors, in the pathway from age at menarche to endometrial cancer incidence.

Our study showed that age at menarche (*p* = 1.21e − 05; OR : 0.6852; 95% CI: 0.5784–0.8116), age at menopause (*p* = 0.00098; OR : 1.242; 95% CI: 1.0919–1.4127), and SHBG levels (*p* = 7.4e − 07; OR : 0.5914; 95% CI: 0.4804–0.7281) have independent causal relationships with endometrial cancer. Age at menarche and SHBG levels are negatively associated with the incidence of endometrial cancer, while age at menopause is positively associated with it. Estrogen serves as the primary stimulus for endometrium proliferation [40]. However, uncontrolled proliferation of the endometrium can potentially lead to malignant transformation. Therefore, estrogen is deemed a fundamental causative factor for the development of at least some cases of endometrial cancer [41]. Evidence indicates that prolonged exposure to sex hormones, such as estrogen, may increase the risk of reproductive organ malignancies [42]. Menarche marks the onset of ovulation and the associated hormonal changes in the female body, while menopause signals the conclusion of reproductive capability. Women who experience earlier menarche and later menopause exhibit higher hormone levels and, hence, longer exposure to estrogen over their lifetime [43]. Therefore, later menarche and earlier menopause may reduce the likelihood of endometrial cancer [6], and these findings are consistent with our results. However, some studies support the notion that late menarche is negatively correlated with EC risk, whereas the age at menopause is positively linked to the probability of endometrial cancer [44, 45].

SHBG is a protein that binds to sex hormones such as estrogen and testosterone. SHBG is synthesized in the liver, which serves as an integrative marker of metabolic and endocrine health. Lower SHBG levels, often seen in obesity and insulin resistance, lead to higher bioavailability of estrogen, thereby promoting endometrial proliferation. This positions SHBG within a key endocrine‐metabolic cross‐talk that directly influences endometrial cancer risk. SHBG levels are affected by the menstrual cycle. During the follicular phase of the menstrual cycle, estrogen levels rise and stimulate the production of SHBG. During the luteal phase of the menstrual cycle, progesterone levels rise and can counteract the effects of estrogen on SHBG production. As a result, SHBG levels may decrease during this phase, which can increase the bioavailability of sex hormones in the body. Previous studies have suggested an inverse correlation between SHBG and endometrial cancer, which is consistent with the findings of this study [46]. The reason may be that SHBG binds to estrogen and reduces its bioavailability and limits its ability to stimulate the proliferation of the endometrium.

Previous studies have suggested that periods may affect the occurrence of endometrial cancer by influencing levels of estrogen and progesterone, which are closely related to human metabolism [47, 48]. Other research has also indicated that metabolic factors can affect the development of endometrial cancer [49–51]. Therefore, we analyzed the mediating effects of 19 factors, including fat metabolism, sugar metabolism, protein metabolism, and obesity‐related traits. We found that BMI, obesity, waist circumference, body fat percentage, and waist‐to‐hip ratio play important mediating roles in the causal relationship between age at menarche and endometrial cancer. It is worth noting that these mediating factors are all obesity related. Previous studies have shown that excessive obesity can promote endometrial cancer through estrogen secreted by adipocytes and the inflammatory response mediated by adipocyte‐secreted adipokines [52–53]. Our study also found a positive correlation between obesity‐related factors and endometrial cancer. Therefore, we hypothesize that for women with early menarche or late menopause, preventing obesity may be a way to prevent endometrial cancer. However, as these traits are highly genetically correlated, their individual mediation proportions should not be considered entirely independent and may reflect overlapping biological pathways. Our use of MVMR in the second step helps to isolate the independent effect of each mediator conditional on the exposure, but residual uncertainty due to shared biology remains [52].

This study has several strengths. First, it is the first study to use MR analysis to explore the causal relationship between period‐ and pregnancy‐related factors with endometrial cancer. Second, the study employs MVMR, which allows for a robust interpretation of the independent effects of exposures on endometrial cancer. Third, the study uses the two‐step MR for the first time to explore the mediating factors underlying the causal relationship between period‐related factors and endometrial cancer and finds that obesity‐related traits play an important mediating role, providing new insights for the prevention and treatment of endometrial cancer.

However, our study also has limitations. First, due to data resource limitations, not all data related to mediating factors are from women. Second, this study uses multiple methods for sensitivity analysis, and there is heterogeneity in SNPs used for age at menarche and age at menopause, but we used a random effects model to confirm the causal relationship between these exposures and endometrial cancer. Additionally, in this study, variables sourced from the UKB or FinnGen databases were utilized as exposures, and data published by O′Mara et al. were used as outcomes, following the principles of two‐sample MR. Unfortunately, we were unable to find additional high‐quality endometrial cancer data apart from the UKB, FinnGen, and O′Mara datasets within the IEU database, GWAS databases, and relevant literature. Although we employed pleiotropy‐robust methods (MR Egger and weighted median) and outlier removal (MR‐PRESSO), residual horizontal pleiotropy via pathways such as inflammation or puberty timing cannot be entirely ruled out. Steiger directionality tests confirmed the assumed causal direction. Our sensitivity analyses suggest these biases are minimal, but results should be interpreted with this inherent limitation in mind. And a key limitation of our study is the reliance on GWAS data from European ancestry populations, which limits the generalizability of our findings. Future replication in multiancestry cohorts is essential to validate these causal relationships and explore potential transethnic differences.

## 5. Conclusions

In summary, this MR study elucidates the independent effects of period‐ and pregnancy‐related factors on endometrial cancer and explores the mediators. We find that obesity‐related traits, including BMI, obesity, waist circumference, body fat percentage, and waist‐to‐hip ratio, play an important mediating role in the relationship between age at menarche and endometrial cancer. This study provides causal evidence for the etiology of endometrial cancer and offers new ideas for its prevention, providing new avenues for further research.

## Disclosure

All authors read and approved the final manuscript.

## Conflicts of Interest

The authors declare no conflicts of interest.

## Author Contributions

Meifang Zhou: conception, study design, data collection and analysis, and manuscript writing/editing; Suiping Dai: data collection and manuscript writing/editing; Tingting Zhu: manuscript editing; Shihao Hong: manuscript linguistic embellishment. Meifang Zhou and Suiping Dai contributed equally to this work and share the first authorship.

## Funding

No funding was received for this manuscript.

## Supporting Information

Additional supporting information can be found online in the Supporting Information section.

## Supporting information


**Supporting Information 1** Table S1: The causal association of exposures with endometrial cancer in UVMR.

**Supporting Information 2** Table S2: Pleiotropy and heterogeneity test of the URMR of exposures on endometrial cancer and exposures on mediators.

## Data Availability

The data that support the findings of this study are available from the corresponding author upon reasonable request.
