# Supplementary material for: Causal Relationships Between Pregnancy, Menstrual History, and Endometrial Cancer With Mediating Effects of Metabolism‐Related Traits
Source: Hum Mutat. 2025 Dec 18;2025:3401957. doi: 10.1155/humu/3401957 (PMC12714079; doi:10.1155/humu/3401957)
Supplement: Supplementary file 1 — Supporting Information 1 Table S1: The causal association of exposures with endometrial cancer in UVMR. [file HUMU-2025-3401957-s001.docx]

Supplementary Table 1. The causal association of meaningful exposures with Endometrial cancer in univariate MR analysis.

| **Methods** | **Menarche** | **Age at menopause** | **Sex hormone−binding globulin levels** |
| --- | --- | --- | --- |
| IVW | | | |
| OR (95% CI) | 0.6852 (0.8545-0.5158) | 1.242 (1.3708-1.1132) | 0.5914 (0.7994-0.3835) |
| p | 1.21E-05 | 0.001 | 7.40E-07 |
| MR Egger | | | |
| OR (95% CI) | 1.0356 (1.503-0.5681) | 1.3869 (1.648-1.1259) | 0.6751 (1.0414-0.3088) |
| p | 0.8837 | 0.0157 | 0.0362 |
| Weighted median | | | |
| OR (95% CI) | 0.7411 (0.9888-0.4934) | 1.3393 (1.519-1.1595) | 0.6368 (0.954-0.3195) |
| p | 0.0177 | 0.0014 | 0.0053 |
| Simple mode | | | |
| OR (95% CI) | 0.5366 (1.2316-0.1585) | 1.1629 (1.5173-0.8085) | 0.6798 (1.4016--0.042) |
| p | 0.0808 | 0.4058 | 0.2953 |
| Weighted mode | | | |
| OR (95% CI) | 0.6702 (1.2226-0.1178) | 1.3297 (1.532-1.1275) | 0.7558 (1.1777-0.3339) |
| p | 0.1572 | 0.0068 | 0.1941 |
