# Supplementary material for: Causal Relationships Between Pregnancy, Menstrual History, and Endometrial Cancer With Mediating Effects of Metabolism‐Related Traits
Source: Hum Mutat. 2025 Dec 18;2025:3401957. doi: 10.1155/humu/3401957 (PMC12714079; doi:10.1155/humu/3401957)
Supplement: Supplementary file 2 — Supporting Information 2 Table S2: Pleiotropy and heterogeneity test of the URMR of exposures on endometrial cancer and exposures on mediators. [file HUMU-2025-3401957-s002.docx]

Supplementary Table 2. Pleiotropy and heterogeneity test.

|  | **Heterogeneity test** | | | | **[Random effect model](javascript:;)** | | **Pleiotropy test** | |
| --- | --- | --- | --- | --- | --- | --- | --- | --- |
|  | IVW | | MR‐Egger | | *p* | *β* | MR‐Egger intercept | *p* |
|  | Q‐statistics | *p* | Q‐statistics | *p* |  |  |  |  |
| Menarche | 231.8972 | 0.0291 | 227.808 | 0.0394 | 1.21E-05 | -0.3781 | -0.0086 | 0.0649 |
| Age at menopause | 180.5399 | 8.65E-06 | 178.992 | 9.14E-06 | 0.001 | 0.0657 | -0.0054 | 0.3428 |
| Sex hormone−binding globulin levels | 503.2519 | 1.40E-05 | 502.2633 | 1.37E-05 | 7.40E-07 | -0.5252 | -0.002 | 0.3902 |
